# KCNJ2 gene spontaneous missense mutation in a child with Andersen-Tawil syndrome: a case report

**DOI:** 10.3389/fped.2026.1961456

**Published:** 2026-10-12

**Authors:** Meiqi Feng, Xi Wang, Lingshan Hu, Lei Zhang

**Affiliations:** Department of Endocrinology and Metabolism, Shandong Medical and Pharmaceutical University Hospital, Shandong Medical and Pharmaceutical University, Binzhou, Shandong, China

**Keywords:** Andersen-Tawil syndrome, arrhythmia, hypothyroidism, KCNJ2, mutation, periodic paralysis

## Abstract

**Background:**

Andersen-Tawil syndrome (ATS) is a rare disorder featuring ventricular arrhythmias, periodic paralysis, and skeletal anomalies. *KCNJ2* mutations account for about 70% of cases. We report a child with a novel *KCNJ2* mutation and subclinical hypothyroidism, an association not previously described.

**Case presentation:**

A 10-year-old boy presented with episodic limb weakness, palpitations, low-set ears, and bilateral fifth-finger bending. Labs showed spontaneous normokalemia without potassium supplementation, elevated creatine kinase, and subclinical hypothyroidism. ECG revealed frequent ventricular premature beats. Genetic testing identified a heterozygous *KCNJ2* c.461G > A (p.C154Y) mutation, confirmed as *de novo*.

**Results:**

The patient was treated with propafenone and acetazolamide. At six months, paralytic episodes decreased from weekly to one mild event, and premature beats reduced markedly on Holter. No adverse effects occurred.

**Conclusion:**

This case expands the *KCNJ2* mutation spectrum and suggests thyroid screening may be considered in ATS. With proper treatment, short-term prognosis is favorable.

## Introduction

Andersen Tawil syndrome (ATS) was proposed by Tawil and colleagues in 1994. ATS is a rare potassium channel disease involving multiple systems. It presents with autosomal dominant inheritance or scattered individual mutation disease. The prevalence rate is about 1/1,000,000, and 70% are related to the abnormal function of *KCNJ2* gene (1). Kir2.1, encoded by the KCNJ2 gene, is an inward rectifier potassium channel protein mainly expressed in ventricular muscle, skeletal muscle and brain. The abnormal *KCNJ2* gene is characterized by prolonged QT interval and ventricular arrhythmia in myocardial tissue and weakness and periodic paralysis in skeletal muscle (2). Therefore, the typical clinical triad of ATS consists of ventricular arrhythmias, periodic paralysis, and skeletal dysmorphism. In this paper, we present a child patient with ATS caused by spontaneous missense mutation at c.461G > A (p.C154Y) in the *KCNJ2* gene.

## Case presentation

A 10-year-old male presented with periodic weakness of both lower limbs and claudication 7 years ago without special treatment. Over the past few years, the patient's symptoms of muscle weakness in both lower limbs had occurred repeatedly. Five years ago, the patient began to have palpitations. Electrocardiogram (ECG) showed ventricular premature beats, short bursts of ventricular tachycardia. After the treatment of propafenone, the number of premature beats decreased. Since 1 year ago, the patient's periodic lower limb fatigue symptoms have worsened, and his lower limbs were numb and it was even difficult for him to stand. The onset process of the patient is shown in Table 1**.**

**Table 1:** Timeline of the case.

Time	Event
7 years ago	Onset of periodic lower limb weakness and claudication.
5 years ago	Palpitations began; ECG revealed ventricular premature beats and short bursts of ventricular tachycardia; propafenone initiated.
1 year ago	Worsening of periodic lower limb fatigue; numbness and difficulty standing after prolonged sitting.
Current admission	Physical examination: low-set ear lobes, fifth-finger bending deformity, arrhythmia; Laboratory: elevated ALT/AST, subclinical hypothyroidism; Genetic testing: KCNJ2 c.461G > A (p.C154Y).
After treatment	Periodic paralysis relieved; arrhythmia frequency decreased.

We performed a detailed physical examination of the patient. The patient has low earlobe and bending deformity of both hands (Figures 1A,B). Cardiac auscultation showed arrhythmia and premature beats. The muscle strength of lower limbs was grade IV, and the tendon reflex was normal. Ambulatory electrocardiogram showed frequent ventricular premature beats (some showed duplex law and occasionally triple law, Figure 1C). No obvious abnormalities were found in spinal x-ray film, cardiac color Doppler ultrasound, cranial MR and neuroelectromyography. Liver function showed that alanine aminotransferase (ALT) and aspartate aminotransferase (AST) increased slightly (Table 2). Serum potassium was measured on multiple occasions, both at baseline and during episodes of weakness, and all values were within the normal range (4.75 mmol/L on admission; Table 2). The patient did not receive slow-release potassium supplements or any other potassium-modifying agents at any point during the follow-up. Thyroid function suggested sub-clinical hypothyroidism.

**Figure 1:**
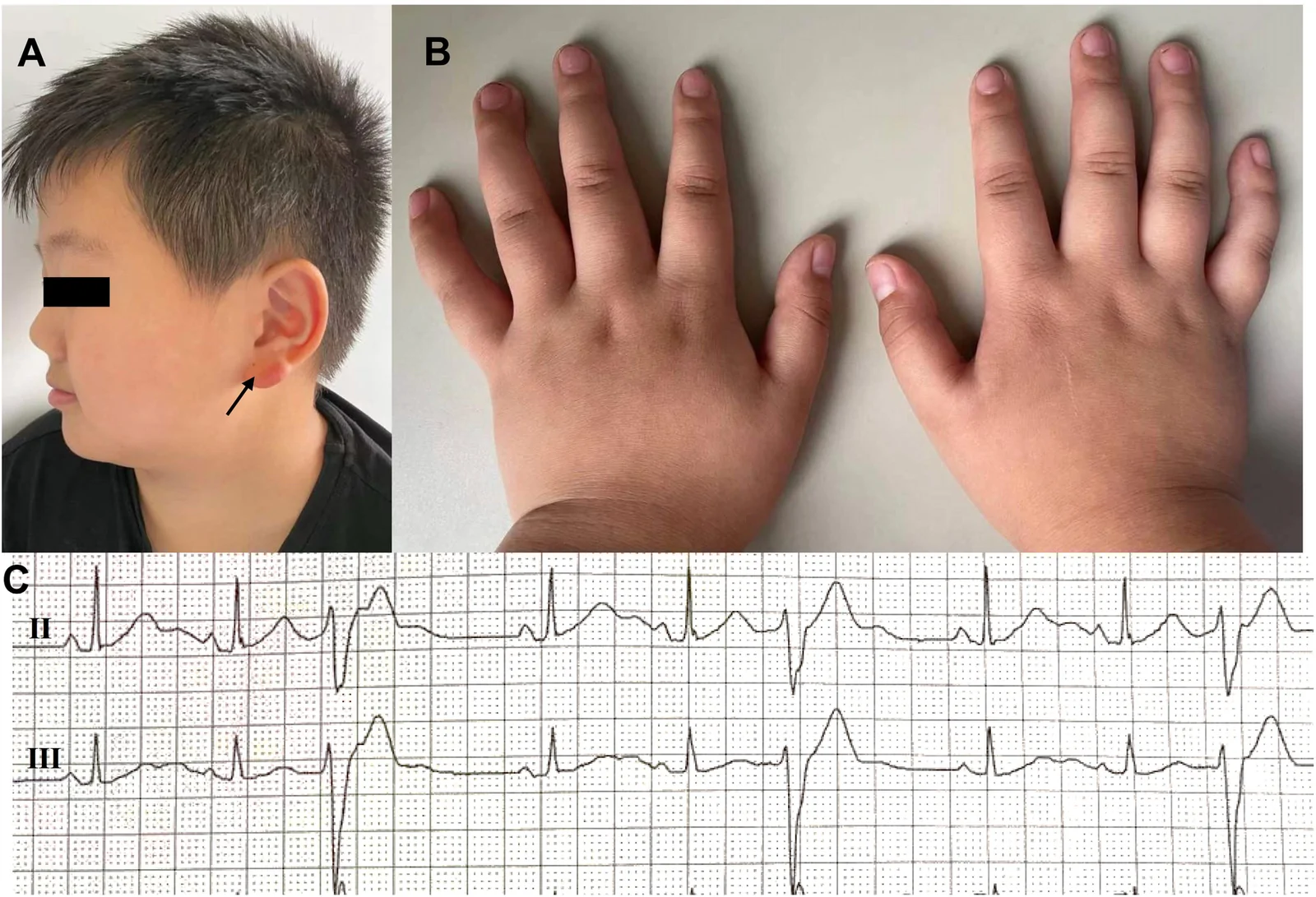
**(A)** the patient has low-set ear lobes. **(B)** The patient's fifth-finger bending deformity. **(C)** The patient's ECG showed ventricular trigeminy.

**Table 2:** Laboratory tests of the patient.

Variable	Test Value	Reference Range
Blood Electrolytes		
Potassium(mmol/L)	4.75	3.5–5.5
Sodium(mmol/L)	137.3	137–147
Chloride(mmol/L)	102.3	99–111
Calcium(mmol/L)	2.56	2.1–2.55
Phosphate(mmol/L)	1.70	0.81–1.45
Magnesium(mmol/L)	0.94	0.7–1
Renal function index		
BUN (mmol/L)	6.75	3.1–8.0
Cr(*μ*mol/L)	32.90	21.5–104
UA(μmol/L)	297.5	208–408
Liver function index		
AST（U/L）	68.80	15–40
ALT（U/L）	105.20	9–50
Thyroid Function		
TSH (mIU/L)	6.010	0.56–5.91
FT3(pmol/L)	7.89	3.28–6.47
FT4(pmol/L)	14.41	7.64–16.03
PTH(nmol/L)	30.46	15–65
Renin Angiotensin Aldosterone System(supine)		
Renin (μIU/mL)	38.26	4.2–45.6
AngiotensinII（pg/mL）	42.14	25–250
Aldosterone（pg/mL）	150.8	70–300

**BUN** blood urea nitrogen; **Cr** creatinine; **UA** uric acid；**AST** Aspartate aminotransferase；**ALT** alanine aminotransferase；**TSH** Thyroid stimulating hormone；**FT3** Free thyroid hormone3；**FT4** Free thyroid hormone4；**PTH** parathyroid hormone.

Finally, genetic testing was performed on the patient and his parents. Heterozygous missense mutation at c.461G > A (p.C154Y) site of *KCNJ2* gene was found in the exon of the patient (Figure 2). There was no variation in this locus in the parents. The protein function prediction software (REVEL, SIFT, PolyPhen-2, Mutation Taster and GERP+) suggested the mutation is harmful. There is no report of this site in the literature database. The patient was treated with propafenone and acetazolamide, the periodic paralysis of lower limbs was relieved.

**Figure 2:**
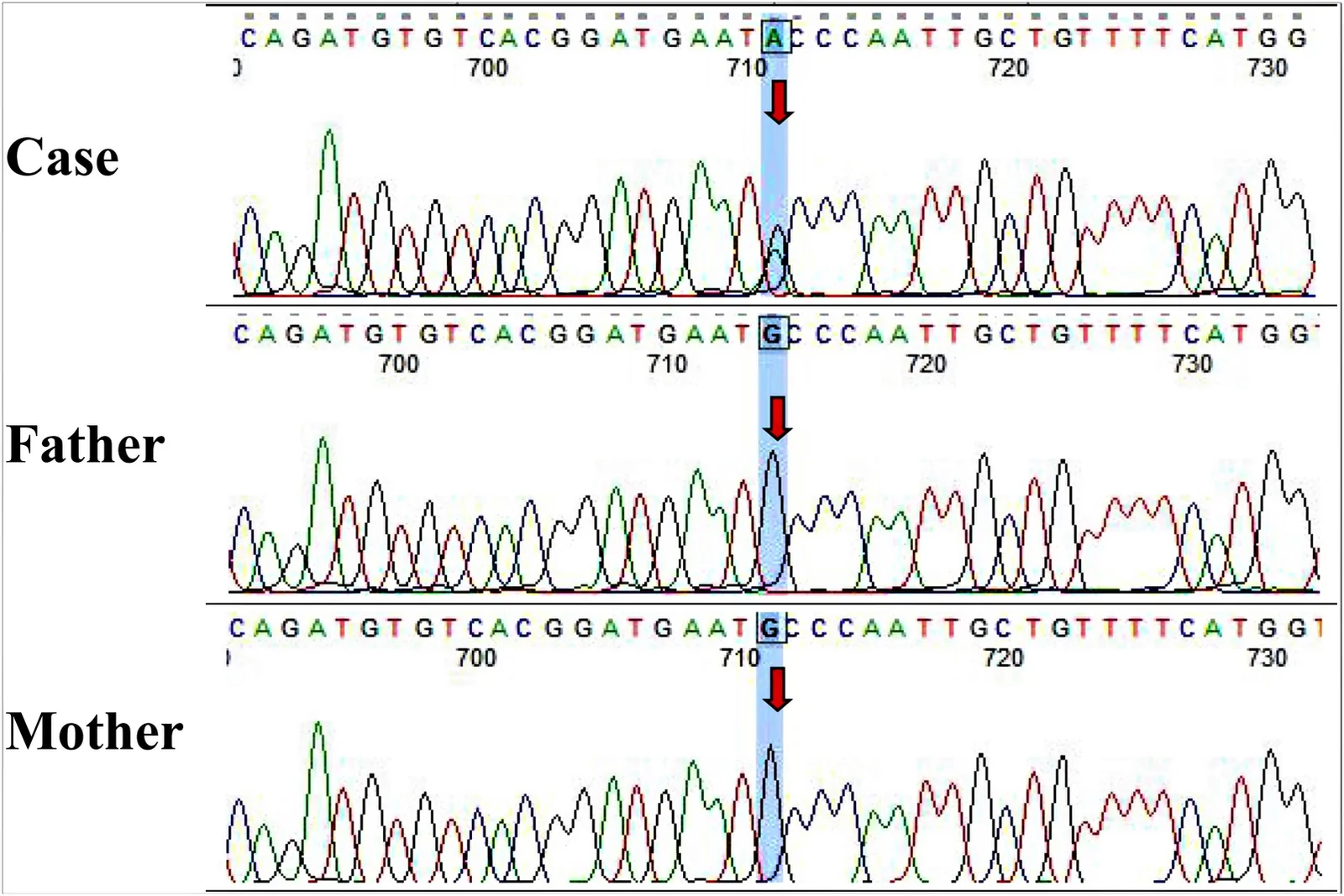
Heterozygous missense mutation at c.461G > A (p.C154Y) site of *KCNJ2* gene in the exon of the case.

## Discussion

ATS cardiac involvement is characterized by functional and structural heart disease, and the most common functional damage is arrhythmia (3). Ventricular arrhythmia is considered to be one of the characteristic changes of ATS cardiac involvement. About 88% ATS patients have ventricular arrhythmia (4). The ambulatory electrocardiogram of this patient showed frequent ventricular premature beats, consistent with previous report.

Periodic paralysis in ATS patients can occur in hyperkalemia, hypokalemia and normal blood potassium. Previous data showed that 64% of ATS patients were complicated with periodic paralysis, of which hypokalemic paralysis accounted for about 65% (5). Normokalemic paralysis in ATS is rare. Our patient was mainly characterized by decreased proximal muscle strength and spontaneous normokalemia in the absence of potassium supplementation. In addition, our patient had elevated creatine kinase and subclinical hypothyroidism, findings that have not been reported previously.

Facial and skeletal developmental malformation is one of the phenotypes to evaluate ATS. Facial malformations can be manifested as deep-set eyes, hypertelorism (increased interocular distance), short palpebral fissures, low ear, etc. Skeletal abnormalities include short stature, small head, scoliosis, etc (6). Congenital digital (toe) scoliosis and mandibular hypoplasia are the most common (5, 7). The severity of malformation does not correlate the degree of skeletal muscle and myocardial involvement. Our patient presented with bending of the fifth finger, but the facial deformity was not typical.

ATS is an ion channel disease, which is related to abnormal gene function. *KCNJ2* and *KCNJ5* gene mutation is the pathogenic genes of ATS (8). At present, ATS is divided into two types. Type 1 is related to *KCNJ2* gene mutation, and type 2 is partially related to *KCNJ5* gene mutation. At present, more than 30 *KCNJ2* gene mutations have been found, most of which are inherited in families. Gene detection in our case showed that there was a heterozygous mutation site, namely c.461G > A (p.C154Y). This mutation was a missense mutation, which substitutes cysteine with tyrosine at amino acid position 154. This mutation has not been reported previously, and it occurred *de novo*.

At present, there is no treatment standard for ATS. Flecainide can effectively inhibit the ventricular arrhythmia of ATS, and it is recommended as the first-choice drug (9). Flecainide and propafenone belong to the same class of antiarrhythmic drugs. In our case, after the application of propafenone, the frequency of arrhythmia decreased, suggesting that the treatment is effective. Implantable cardioverter defibrillator (ICD) should be considered when the patient has pleomorphic ventricular arrhythmia or severe symptoms such as syncope. For the treatment of ATS periodic paralysis, carbonic anhydrase inhibitors acetazolamide and dichlorphenamide can reduce the onset of periodic paralysis (10). Our case is a periodic paralysis with normal blood potassium. The symptoms of periodic paralysis were alleviated after the application of carbonic anhydrase inhibitor.

The prognosis of ATS is unclear. However, it is generally believed that when children with ATS do not have life-threatening arrhythmias such as sudden cardiac death, the prognosis is favorable.

Given the inherent limitations of a single observation, whether subclinical hypothyroidism represents a true phenotypic extension of ATS remains uncertain. Additional cases are needed to confirm our findings.

## Patient perspective

The patient's mother reported that prior to treatment, her son experienced weekly paralytic attacks, each lasting several hours, which prevented him from attending school and participating in daily activities. After initiating propafenone and acetazolamide, the frequency of episodes dropped to only one mild event over six months. The child himself noted that his palpitations became less bothersome and he could now walk to school without difficulty. The family expressed satisfaction with the treatment and commitment to continued follow-up.

## Conclusion

We report a pediatric ATS case with a novel *KCNJ2* mutation, c.461G > A (p.C154Y), which occurred spontaneously and, to our knowledge, has not been previously documented. The patient also had spontaneous normokalemia without potassium supplementation and subclinical hypothyroidism, an association that appears not to have been reported in ATS, though whether this is coincidental or related remains to be determined. Treatment with propafenone and acetazolamide provided satisfactory symptom control. This case reinforces the value of early genetic testing in suspected ATS and suggests that routine thyroid function screening may be worth considering in these patients. In the absence of malignant arrhythmias, the short-term outlook appears favorable with appropriate medical therapy.

## Data Availability

The original contributions presented in the study are included in the article/Supplementary Material, further inquiries can be directed to the corresponding author.
